# SERS Gas Sensors Based on Multiple Polymer Films with High Design Flexibility for Gas Recognition

**DOI:** 10.3390/s21165546

**Published:** 2021-08-18

**Authors:** Lin Chen, Hao Guo, Fumihiro Sassa, Bin Chen, Kenshi Hayashi

**Affiliations:** 1 Graduate School and Faculty of Information Science and Electrical Engineering, Kyushu University, Fukuoka 819-0395, Japan; chen.lin.658@s.kyushu-u.ac.jp (L.C.); guo.hao.097@s.kyushu-u.ac.jp (H.G.); sassa@ed.kyushu-u.ac.jp (F.S.); 2 Chongqing Key Laboratory of Non-linear Circuit and Intelligent Information Processing, College of Electronic and Information Engineering, Southwest University, Chongqing 400715, China; chenbin121@swu.edu.cn

**Keywords:** gas with similar molecular structures, SERS gas sensor, polymer film, gas affinity, PCA analysis, gas recognition

## Abstract

The Surface-Enhanced Raman Scattering (SERS) technique is utilized to fabricate sensors for gas detection due to its rapid detection speed and high sensitivity. However, gases with similar molecular structures are difficult to directly discriminate using SERS gas sensors because there are characteristic peak overlaps in the Raman spectra. Here, we proposed a multiple SERS gas sensor matrix via a spin-coating functional polymer to enhance the gas recognition capability. Poly (acrylic acid) (PAA), Poly (methyl methacrylate) (PMMA) and Polydimethylsiloxane (PDMS) were employed to fabricate the polymer film. The high design flexibility of the two-layer film was realized by the layer-by-layer method with 2 one-layer films. The SERS gas sensor coated by different polymer films showed a distinct affinity to target gases. The principle component analysis (PCA) algorithm was used for the further clustering of gas molecules. Three target gases, phenethyl alcohol, acetophenone and anethole, were perfectly discriminated, as the characteristic variables in the response matrix constructed by the combination of gas responses obtained 3 one-layer and 3 two-layer film-coated sensors. This research provides a new SERS sensing approach for recognizing gases with similar molecular structures.

## 1. Introduction

Surface-Enhanced Raman Scattering (SERS) is a widely used sensing technique in which the Raman signal of molecules adsorbed onto noble metal surfaces (such as silver or gold) can be greatly enhanced [1,2]. The SERS detection technique has significant potential in clinical diagnosis applications [3,4,5,6], owning to its rapid detection, high sensitivity down to single molecules and non-invasive recognition of disease biomarkers according to its specific Raman signature. In the explosive detection [7,8] and environment monitoring fields [9], the SERS technique could realize ultra-trace, real-time detection and efficient discrimination of detection molecules. The rapid and accurate determination of plant volatile organic compounds by the SERS technique is beneficial for indicating the healthy status of plants [10,11]. The instrumentation used in the rapid SERS detection technique is portable, and SERS sensors are inexpensive to prepare. Therefore, the SERS technique could be utilized for analytical chemistry at the point of sample [12]. Integrating graphene oxide with the SERS sensor can improve the sensitivity of sensors, because graphene oxide/silver composites can generate strong chemical enhancement [13]. Due to the unique vibration frequency of molecules, rich fingerprint information is provided in the Raman spectrum. By processing information from the Raman spectrum, it is possible to effectively accomplish the classification and recognition of samples detected by the SERS sensor [14,15,16]. However, the characteristic peaks of detection samples with similar molecular structures may overlap in the Raman spectra. Therefore, it is necessary to enhance the selectivity of the SERS gas sensor in this detection situation.

Molecularly imprinted polymers (MIPs) are prepared using the target molecules as templates to produce receptor sites. After removing the template, the target molecules can be selectively captured [17,18,19,20,21]. Castro-Grijalba et al. developed a SERS sensor through spin-coating MIP to form thin film for polycyclic aromatic hydrocarbons (PAHs) detection [22]. The selectivity of the MIP-based SERS sensor was realized when three PAHs of different sizes were detected. Another technology, metal-organic frameworks (MOFs), has recently been proposed for improving the selectivity of the SERS sensor [23,24,25,26], which is achieved through the tunable pore size of MOFs [27,28], as well as electrostatic [29] or hydrophobic [30] interactions between the molecules and MOFs. However, the procedures of preparing MIP and MOFs are complex and time-consuming. Besides, as a simple detection method, the polymers are utilized in sensor devices because of their chemical and physical properties [31,32]. For example, PDMS was used to improve temperature sensitivity in the Plasmon Resonance sensor [33]. Moreover, SERS arrays with surfaces modified by different functional materials could be utilized for improving the discriminatory accuracy of specific targets or detecting multiplex target analysts simultaneously. Molly M. Stevens et al. developed an artificial-nose inspired label-free SERS sensor arrays, consisting of eight different self-assembled monolayers (SAMs), diversifying the SERS fingerprints of analytes. Improvements in the classification accuracy of two cell lysates (Hs578Bst and Hs578T) toward 100% were realized using combinations of fingerprints obtained from different SAM-modified SERS sensor arrays [34]. The distinct SERS nanotags conjugated with different antibodies were assembled on nanoparticles for fabricating SERS sensor arrays on one substrate, which were utilized as a rapid and efficient technique for simultaneous multiplex proteins detection [35,36].

**Figure 1:**
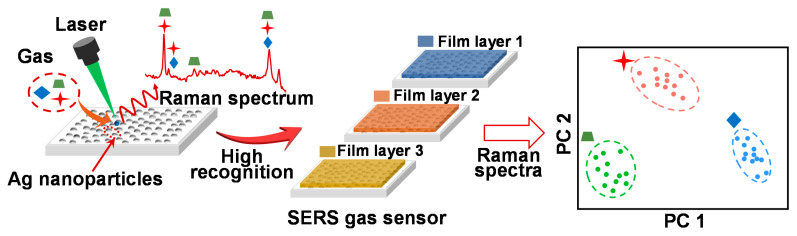
The schematic graph of the polymer film array for gas recognition.

Herein, we propose a method of uncomplicated fabrication and wide-ranging application for constructing a SERS sensor array with different gas affinities, which was spin-coated by distinct functional polymer films for the recognition of gases with similar structures (Figure 1). Functional two-layer polymer film used in the sensor array, which possessed a distinctive gas response, was simply and efficiently fabricated with high design flexibility using the layer-by-layer method. Gas recognition performance was improved by increasing the number of characteristic variables in the response matrix processed by the PCA algorithm. Three plant-based essential oils molecules with similar sizes and structures—phenethyl alcohol [37], acetophenone [38] and anethole [39]—were selected as target gases. Three kinds of polymers possessing different molecular structures and functional groups—Poly (acrylic acid) (PAA), Poly (methyl methacrylate) (PMMA) and Polydimethylsiloxane (PDMS)—were employed as sensing films. One-layer film and two-layer film were prepared by the spin-coating method on the SERS gas sensor. The two-layer film with high design flexibility was fabricated using the layer-by-layer method. The diverse gas responses were obtained when the three gases were detected by the different polymer film-coated sensors. A response matrix constructed by the Raman intensities of target gases was processed using the PCA algorithm. The characteristic variables of response matrix were tuned using the Raman intensity collected from different sensors to optimize the performance of gas recognition. The purpose of this study is to propose a widely, commonly used SERS gas sensor-based strategy for the recognition of gases with similar structures.

## 2. Materials and Methods

### 2.1. Materials and Instrumentation

Phenethyl alcohol was purchased from Kanto Chemical CO., INC. (Tokyo, Japan). Anethole was purchased from Nacalai TESQUE, INC. (Tokyoto, Japan). Acetophenone, Poly (acrylic acid) (PAA), Poly (methyl methacrylate) (PMMA), ethanol, chloroform and hexane were purchased from Wako Pure Chemical Industries CO., Ltd. (Osaka, Japan). Polydimethylsiloxane (PDMS) was purchased from Shin-Etsu Chemical CO., INC. (Tokyo, Japan). All reagents were used as received. RandaS SERS sensor was purchased from ATOID CO. (Japan). Fourier-transform infrared spectroscopy (FT/IR-6800, Jasco, Japan) was used to analyze the polymer film coated on the SERS substrate. Scanning electron microscopy (SEM, SU8000, Hitachi, Japan) was used to identify the image sensor morphologies. Raman spectra were obtained by a Raman spectrometer (AvaRaman, Avantes, Japan), with a 532 nm excitation laser as a light source (power = 1.5 mW). The accumulation time was set as 10 s for data collection.

### 2.2. Polymer Solution Preparation

Three polymer (PAA, PMMA, PDMS) solutions were prepared. Briefly, PAA (30 mg) was dissolved in ethanol (16 mL), PMMA (30 mg) was dissolved in chloroform (16 mL) and PDMS (2 mg) was dissolved in hexane (16 mL). The prepared polymer solutions were fully stirred before use.

### 2.3. Fabrication of Polymer-Coated SERS Sensor

First, SERS substrate was cleaned by immersion in acetone, ethanol and ultrapure water sequentially. Then, the substrate dried using nitrogen stream. The SERS sensor without the spin-coated polymer film was used as a bare SERS sensor. Th spin-coating method was used to fabricate the SERS gas sensors with polymer film. For the one-layer polymer film-coated SERS sensor, the polymer solution (15 uL) was spin-coated on the SERS substrate and then dried in a vacuum. Spin-coating was performed at 500 rpm for 20 s, a slope of 500 rpm to 3000 rpm for 10 s and 3000 rpm for 30 s. The two-layer polymer film-coated SERS sensor was fabricated using the layer-by-layer method. The first polymer film was prepared on the cleaned SERS substrate, and after the first film was dried, the second polymer film was spin-coated.

### 2.4. Gas generation and Detection System

**Figure 2:**
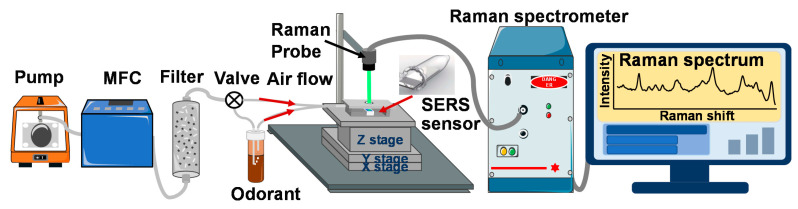
Schematics of the gas generation and detection system.

The schematic diagram of the gas generation and detection system is shown in Figure 2. The flow rate of carrier gas generated from air pump was set to 0.1 mL/min using a mass flow controller (MFC). A gas-cleaning filter was connected between the MFC and a glass bottle with odorant (3 mL). The cleaning filter used was filled with molecular sieve and zeolite to control the humidity level of the carrier gas. The target gas was generated using the bubbling method and then detected in the homemade gas detector cell. The gas concentrations were calculated by [40]:

(1) $$C\;=\;\frac{{k\;\times \;D}_{r\;}{\times \;10}^{3}}{F}$$

where *D_r_* is the diffusion rate (ug/min), *F* is the flow rate of the dilute air (0.1 L/min) and *k* is the factor used to convert gas weight to volume, calculated by:

(2) $$k\;=\;\frac{22.4\;\times \;(273\;+\;t)\;\times \;760}{M\;\times \;273\;\times \;P}$$

where M is the molecular weight, *t* is the temperature in the gas detector cell (20 °C) and P is the gas pressure (760 mmHg). The phenethyl alcohol, acetophenone and anethole concentrations of the target gas were calculated to be 403.4 ppm, 510.2 ppm and 623.7 ppm, respectively.

A metallic cover was designed to prevent laser leakage, and an adjustable stage was manufactured to adjust the position and size of the laser focused on the SERS gas sensor. Raman spectra of the three target gases were recorded after the SERS gas sensor was exposed to each gas for a certain time. Besides, the gas detector cell was washed with air flow for 1200 s after gas detection.

## 3. Results

### 3.1. Raman Spectra of the Detected Gases

**Figure 3:**
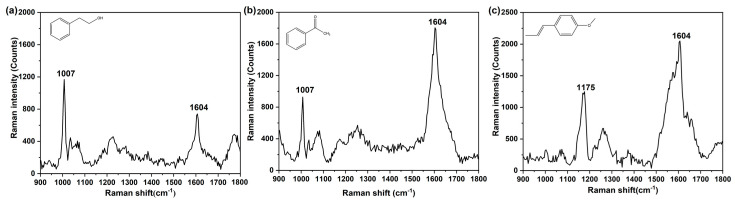
Raman spectra of (**a**) phenethyl alcohol gas, (**b**) acetophenone gas and (**c**) anethole gas.

The morphologies of the sensors are shown in Figure S1. The size of the Ag nanoparticles was around 50 nm (Figure S1a). The results showed that Ag nanoparticles gathered and formed a cluster which could produce more hotspots. Phenethyl alcohol, acetophenone and anethole were selected as the target gases, owing to their similar molecular structure with a benzene ring. The Raman spectra of the three target gases were obtained using a bare SERS sensor (Figure 3). For phenethyl alcohol and acetophenone, the characteristic peaks at 1007 cm^−1^ and 1604 cm^−1^ were assigned to the ring breathing vibration and ring stretching mode [41] (Figure 3a,b), respectively. As shown in Figure 3c, the benzene ring stretching vibration at 1604 cm^−1^ and C-O-C stretching vibration at 1175 cm^−1^ of anethole gas could be clearly observed [42]. The main characteristic peaks of the target samples detected were similar in the gaseous and liquid states. However, when compared with the Raman spectra of these three target analytes solutions (Figure S2), the characteristic peaks of the gases were lower, with weaker Raman intensities. The reason might be due to the difficulty of gas adsorption on the surface of the bare SERS sensor. There was a distinct characteristic peak at 1175 cm^−1^, which could be used to distinguish the acetophenone and phenethyl alcohol gases. However, the two apparent peaks at 1007 cm^−1^ and 1604 cm^−1^ were both observed in the Raman spectra of acetophenone and phenethyl alcohol gases. Thus, it is difficult to directly distinguish these two target gases only through the position where the peaks appeared.

### 3.2. FT-IR Spectra of Functional Polymer Film

As the characteristic peaks in the Raman spectra of the phenethyl alcohol and acetophenone gases were similar, it was difficult to distinguish these two gases through the Raman spectra. However, it is well known that the polarity, size and molecular structure, along with many other properties of polymers, are distinctive. Consequently, polymers are utilized to fabricate gas sensors [31]. Herein, we propose the method of changing the affinity of the SERS gas sensor by fabricating polymer film on the sensor, using to spin-coating method to identify different gases with similar molecular structures.

**Figure 4:**
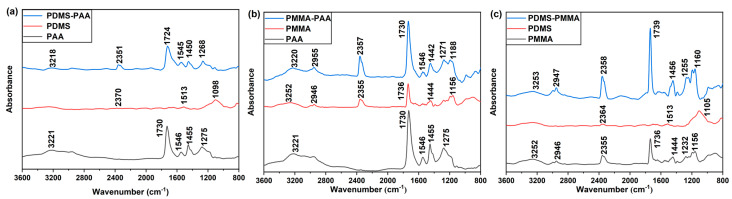
FT-IR spectra in the 800–3600 cm^−1^ range of the SERS gas sensor coated by (**a**) PDMS-PAA, (**b**) PMMA-PAA and (**c**) PDMS-PMMA polymer film.

Three kinds of polymers with different molecular structures and polarities—PAA [43], PMMA [44] and PDMS [45]—are widely employed as functional polymer films of gas sensors. Here, these polymers were utilized in our designed SERS gas sensors. Besides, we designed two types of functional polymer films. In particular, one-layer and two-layer polymer film were prepared to conduct our experiment. The two-layer film was made of 2 one-layer films using the layer-by-layer method. With this method, functional two-layer polymer film with high design flexibility was realized by selecting two different one-layer films according to the detection requirements. For the two-layer polymer film sensor, if polymer B was spin-coated on polymer A, then the sensor was called the A-B SERS gas sensor, i.e., the PDMS-PAA SERS gas sensor was fabricated by spin-coating PAA polymer film on PDMS film. According to similar spin-coating conditions as used in our previous study, the thickness of the prepared film layer in this work was lower than 10 nm [46]. As shown in Figure 4, the FT-IR spectra were obtained using the reflection absorption spectroscopy method in the range of 800–3600 cm^−1^ recorded for one-layer and two-layer polymer films. The bending vibrations of the PAA [47], PMMA [48] and PDMS [49] polymers were confirmed by relative reference. In this experiment, we first fabricated the first polymer film on the bare SERS sensor. Then, we collected the FT-IR spectrum. Second, the second polymer film was spin-coated on this sensor, and the FT-IR spectrum was recorded again. According to the spectra characteristics, we confirmed that the polymer films were attached to the SERS sensor. Besides, we found that only the bending vibration of the top polymer film appeared when a two-layer film SERS sensor was detected, which proves that the first polymer film layer was almost completely covered by the top polymer film layer.

### 3.3. Sensitivity of Polymer Film-Coated SERS Gas Sensor

**Figure 5:**
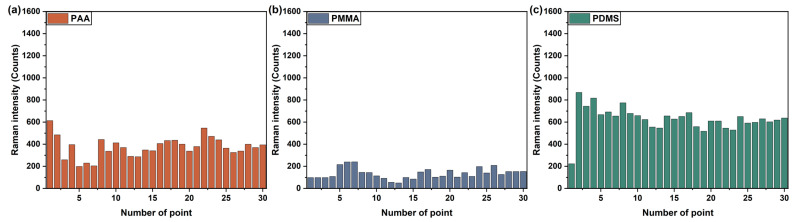
Raman intensity collected from the random 30 points obtained using the (**a**) PAA, (**b**) PMMA and (**c**) PDMS polymer film-coated SERS gas sensor for phenethyl alcohol gas detection.

The background Raman spectra of the bare substrate, PAA-, PMMA- and PDMS-coated sensors were collected, as shown in Figure S3. There were no characteristic peaks at 1006 cm^−1^, 1175 cm^−1^ or 1604 cm^−1^ in the background Raman spectra. All Raman spectra were obtained after subtracting the baseline of the SERS sensor. The phenethyl alcohol, acetophenone and anethole gases were detected by polymer film-coated SERS gas sensors, respectively. The target gas was exposed to the sensor before Raman spectra were collected. It is known that regions on the SERS sensor have hotspots with different signal enhancements [50]. Hence, point-to-point reproducibility across a SERS senor is vital to precisely assess the sensitivity of the sensor. In this work, the Raman spectra of 30 points were randomly obtained from the polymer film-coated SERS gas sensor. Besides, the Raman intensities of phenethyl alcohol gas at 1007 cm^−1^, as detected by the PAA-coated, PMMA-coated and PDMS-coated sensors, were calculated and plotted in Figure 5. The PMMA-coated and PDMS-coated sensor showed the lowest and highest sensitivity for phenethyl alcohol gas, respectively. The Raman spectra of the phenethyl alcohol gases obtained from the polymer film-coated sensors are shown in Figure S4. Moreover, the Raman intensity results of acetophenone gas at 1007 cm^−1^, and anethole gas at 1175 cm^−1^, as detected by the three polymers film-coated SERS gas sensors, are shown in Figures S5 and S7, respectively. The Raman spectra of the acetophenone and anethole gases obtained from the polymer film coated sensors are illustrated in Figure S6 and Figure S8, respectively. Due to the varying affinities of the physicochemical properties of polymers for the target gas, the amount of gas absorbed on the sensor surface was different, which induced the diversity of intensity in the Raman signal for the target gas. In addition, a small variation of the points in Raman intensities was observed on one sensor. There are two main reasons for the variation of Raman intensity on the SERS gas sensor: (1) The density and size of Ag nanoparticles sputtered on the SERS substrate had certain uniformity. Therefore, the intensities and quantities of the hotspots were inhomogeneous. (2) Due to the fluidity of the gas, the attachment point of gas on the sensor was indefinite. Hence, the quantity of gas on the detection point was not same. Thus, the gathered 30 points were used in the data analysis to improve the accuracy.

**Figure 6:**
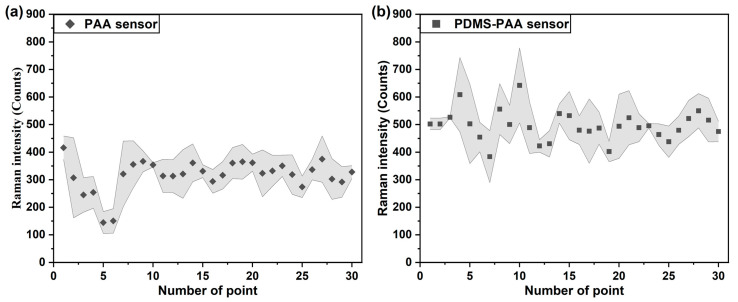
Error band calculated using 30 points obtained from (**a**) PAA-coated and (**b**) PDMS-PAA-coated SERS gas sensor for phenyl ethanol gas detection.

Batch-to-batch producibility is another evaluation parameter for the SERS sensor [51]. As the different gas responses in the one-layer polymer film sensors were definite, the gas response of the two-layer polymer film sensor was also investigated. Here, phenethyl alcohol gas was detected by the PAA-coated and PDMS-PAA-coated SERS gas sensor. The same relative positions of 30 detected points to the gas inlet on the three SERS gas sensors were set by adjusting the x-y stage prepared in the detection system. It takes longer for the gas molecules to adhere to the surface of the SERS gas sensor because the two-layer polymer film is thicker than the one-layer polymer film. The average values and error bands of the same relative position points (three points) on three sensors were calculated, as shown in Figure 6. Though the Raman intensities of the 30 points showed variation, the error bands at the same position were relatively narrow, which indicates that the batch-to-batch producibility of the SERS gas sensor was acceptable. Besides, the Raman intensity of phenethyl alcohol gas detected by the PDMS-PAA-coated sensor was higher than that of the PAA-coated sensor. Compared with the PAA-coated sensor, the PDMS polymer had a better affinity to the phenethyl alcohol gas (Figure 5). Thus, the two-layer PDMS-PAA polymer film structure improved the affinity of the sensor to the phenethyl alcohol gas. Hence, we can confirm that a new response property can be achieved by designing two-layer polymer film with 2 one-layer polymer films. Three kinds of two-layer polymer film, PDMS-PAA, PMMA-PAA and PDMS-PMMA, were chosen to fabricate the SERS gas sensor to detect the phenethyl alcohol, acetophenone and anethole gases.

### 3.4. Gas Recognition by a One-Layer Polymer Film-Coated SERS Gas Sensor Array

#### 3.4.1. Target Gas Recognition by One-Layer Polymer Film-Coated Sensor

As the Raman intensity results of one characteristic peak described before (Figure 5, Figures S5 and S7), the polymers used in this study showed different affinities to the three target gases. Instead of the intensity value at one characteristic peak, the Raman intensities at 1007 cm^−1^, 1175 cm^−1^ and 1605 cm^−1^ were utilized as the response to discriminate the three target gases. First, a one-layer polymer film SERS gas sensor was used to achieve the recognition of three target gases. In total, 90 samples (3 target gases × 30 points in 1 detection) were set as the total sample, and the intensity values in the three selected peaks were considered as the characteristic variables. Hence, a response matrix $M_{90\times 3}$ was obtained for the gas recognition analysis. PCA is an unsupervised machine learning method for the dimensionality reduction of high-dimensional data [52,53]. Principal components (PCs) were extracted from the linear combinations of the original variables. Then, the cluster trends of the target gases were visualized in a two-dimensional space utilizing the two uncorrelated PC scores. In this work, the response matrix was preprocessed by autoscaling to reduce the large variation of different Raman intensity dates. The two dominant PC scores (PC1 and PC2) were used to plot the two-dimensional graphs. The samples were clustered together based on their PC scores related to Raman intensities at different characteristic peaks.

**Figure 7:**
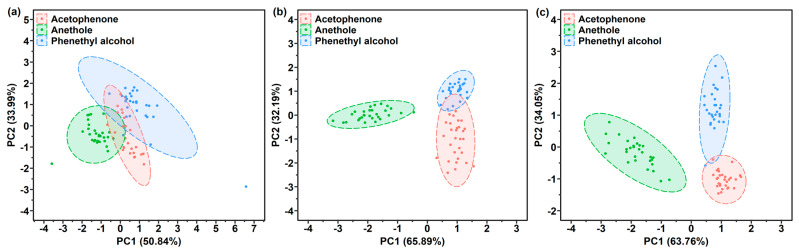
PCA score plots by (**a**) PAA-coated, (**b**) PMMA-coated or (**c**) PDMS-coated SERS gas sensor used for the gas detection of phenethyl alcohol, acetophenone and anethole.

PCA score plots of the samples, as detected by the bare sensor, are shown in Figure S9. As can be seen in Figure S9, the clusters of phenethyl alcohol and acetophenone had a cross section, while the cluster of anethole could be well recognized. These results might be attributed to the similarity of the Raman spectra of these two gases, which showed common characteristic peaks at 1007 cm^−1^ and 1605 cm^−1^, while the peak of anethole was observed at 1175 cm^−1^. Hence, the anethole gas could be discriminated from the phenethyl alcohol and acetophenone gases. Besides, a horizontal distribution of these three clusters was found, which indicates that the Raman intensity at 1175 cm^−1^ plays important role in PC1. The distribution of 90 samples collected from a one-layer polymer film sensor is plotted in the PC1-PC2 space, as shown in Figure 7. In general, the clusters of three gases presented different distribution patterns. The clusters of the 90 samples detected by the PAA-coated sensor overlapped with each other, and the clusters in the PC1-PC2 space of the PMMA-coated and PDMS-coated sensor were distributed both horizontally and vertically. The phenethyl alcohol cluster overlapped with the acetophenone cluster, as shown in Figure 7a–c. A phenethyl alcohol sample was wrongly classified into the acetophenone cluster, as shown in Figure 7c, which can be attributed to their similar Raman spectra. The distribution patterns in the three polymer-coated sensor space confirm that these three polymers possess different gas responses for phenethyl alcohol, acetophenone and anethole. However, the three target gases did not occupy completely separate regions.

#### 3.4.2. Target Gas Recognition of the 2 One-Layer and 1 Two-Layer Polymer Film-Coated SERS Gas Sensors

**Figure 8:**
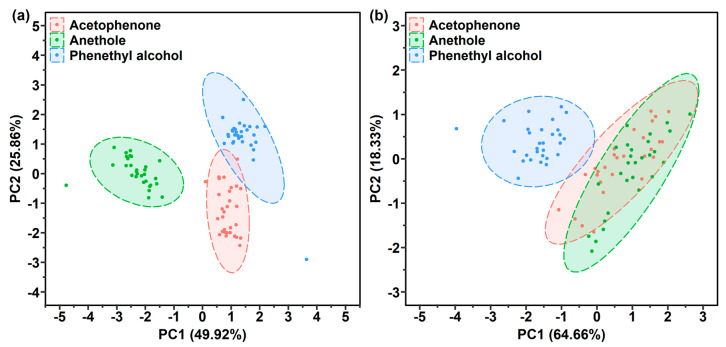
Comparison of PCA score plots of the (**a**) PDMS-coated and PAA-coated SERS gas sensors combination, and (**b**) one PDMS-PAA-coated SERS gas sensor used for the gas detection of phenethyl alcohol, acetophenone and anethole.

Since three kinds of target gases could not be recognized by the one-layer polymer-coated SERS gas sensor, the gas responses obtained from two kinds of polymers were used to conduct the gas recognition. The responses of two polymers can be collected two different ways: the Raman intensities recorded from (1) combinations of 2 one-layer polymer film-coated sensors, or (2) 1 two-layer polymer film-coated sensor. If the gas response of polymer A and B is defined as R_A_ and R_B_, then the gas response of the two-layer polymer A-B might be R_A_ + R_B_ (the combination of the responses of A and B) or R_AB_ (a new gas response characteristic). Herein, the PCA method was utilized to determine the method with better recognition ability for the three target gases. The response matrix $M_{90\;\times \;6}$ of the 2 one-layer polymer film-coated sensors combination was constructed using 90 samples (3 target gases × 30 points) and 6 characteristic variables (3 Raman intensities × 2 polymer films). In addition, the response matrix $M_{90\times 3}$ of the two-layer polymer film-coated sensor consisted of 90 samples and 3 characteristic variables (3 Raman intensities detected by 1 sensor). The PCA score plots of the PAA-coated and PDMS-coated SERS gas sensor combination and PDMS-PAA-coated sensor are plotted in Figure 8. As shown in Figure 8a, the anethole cluster was distributed in a separate space. However, a relatively small overlap appeared between the phenethyl alcohol and acetophenone cluster. Besides, an acetophenone sample appeared in phenethyl alcohol cluster, which indicates that the phenethyl alcohol and acetophenone clusters could not be completely discriminated by the PAA-coated and PDMS-coated sensor combination. The PDMS-PAA-coated sensor showed a worse recognition performance, as shown in Figure 8b. The recognition performance of the PMMA-coated and PAA-coated sensor combination was better than the PMMA-PAA-coated sensor, as shown in the PCA score plots in Figure S10. As expected, the three gas clusters were well differentiated by the response combinations of the PDMS-coated and PMMA-coated sensors (Figure S11). However, the distance between the anethole cluster and phenethyl alcohol cluster was relatively close. It may be possible to increase the distance between the three gas clusters by increasing the number of characteristic variables in the response matrix. In summary, the two-layer polymer film-coated sensor presented a new response characteristic when compared with the one-layer polymer film-coated sensor. More importantly, compared with the fabricated 1 two-layer polymer film-coated sensor, the combination of the responses of the 2 one-layer polymer film-coated sensors showed better gas recognition performance.

#### 3.4.3. Gas Recognition Ability Improvement by Increasing Characteristic Variables in Response Matrix

**Figure 9:**
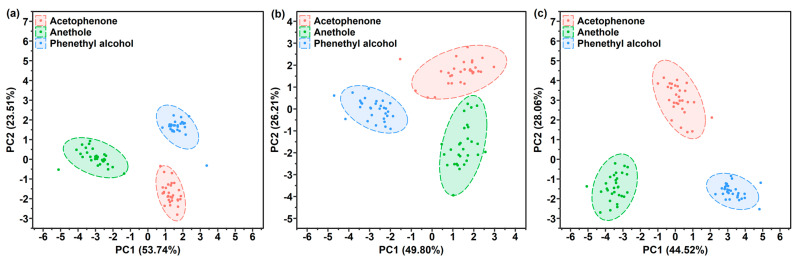
PCA score plots of the (**a**) 3 one-layer polymer film-coated SERS gas sensors, (**b**) 3 two-layer polymer film-coated SERS gas sensors and (**c**) 3 one-layer and 3 two-layer polymer film-coated SERS gas sensors used for phenethyl alcohol, acetophenone and anethole gases detection.

The response matrix constructed by the gas responses obtained from the multiple polymer film-coated SERS gas sensors was applied for gas recognition. Consequently, three response matrices $M_{90\times 9\;}$ (3 one-layer film-coated sensors), ${M^{\prime }}_{90\times 9}\;$ (3 two-layer film-coated sensors) and $M_{90\times 18}\;$ (a combination of 3 one-layer and 3 two-layer film-coated sensors) were obtained. Concretely, $M_{90\times 9}\;$ (3 one-layer film-coated sensors) and ${M^{\prime }}_{90\times 9}\;$ (3 two-layer film-coated sensors) were equally constructed by 90 samples and 9 characteristic variables (Raman intensities at 3 characteristic peaks × 3 polymer films). The number of characteristic variables of $M_{90\times 18}$ was set at 18, which consisted of Raman intensities at 3 characteristic peaks × (3 one-layer + 3 two-layer polymer films). PCA score plots in the PC1-PC2 space of these three response matrices are shown in Figure 9. By increasing the number of characteristic variables from six to nine, three gas clusters were completely separated, as shown in Figure 9a. In addition, the distance of the clusters grew more similar to the result shown in Figure S11. As response matrix ${M^{\prime }}_{90\times 9}\;$ (two-layer film) had the same number of characteristic variables as $M_{90\times 9}\;$ (one-layer film), three gas clusters could be easily recognized. In contrast, the anethole cluster was close to the acetophenone cluster, as plotted in Figure 9b. The two-layer polymer film-coated sensor presented a new response property for the three target gases. Thus, the same amount of information was provided as the one-layer polymer film-coated sensor. It is noteworthy that the three gas clusters occupied a far-distance distribution space, as shown in Figure 9c. Hence, more characteristic variables in the response matrix processed by the PCA method could effectively enhance the ability of gas recognition based on our results.

## 4. Discussion

In summary, multiple polymer film-coated SERS gas sensors were developed to distinguish gases with similar structures. Three gases consisting of a benzene ring and distinctive functional groups—phenethyl alcohol, acetophenone and anethole—were selected as target gases. PAA, PMMA and PDMS were employed to fabricate one-layer polymer films on the SERS gas sensors using the spin-coating method. Moreover, two-layer polymer film could be flexibly designed with 2 one-layer films using the layer-by-layer method. The Raman spectra of three target gases were obtained when they were detected by polymer-coated sensors with different gas affinities. Raman intensities at the selected characteristic peaks of target gases were used as characteristic variables in the response matrix for PCA analysis. Besides, characteristic variables could be changed using the Raman intensities detected by the different polymer film-coated sensors. It can be concluded from the PCA results that gas molecules with similar structures were accurately discriminated, and the two-layer film-coated sensor presented a new response property compared to the one-layer film-coated sensor. The best gas recognition result was observed when the response matrix was constructed by 18 characteristic variables according to the PCA score plot. Moreover, the gas recognition performance was improved by increasing the number of characteristic variables in the response matrix. This study verifies that the recognition of gases with similar molecular structures can be realized by SERS sensors spin-coated with multiple polymers possessing different affinities.

## Data Availability

Not applicable.

## Supplementary Materials

The following are available online at https://www.mdpi.com/article/10.3390/s21165546/s1, Figure S1: (a) SEM images of Ag nanoparticles (b) EDX results of selected area obtained from bare substrate, Figure S2: Raman spectra of (a) Phenylethyl alcohol, (b) Acetophenone and (c) Anethole solution detected by bare SRES substrate, Figure S3: The background Raman spectra of bare substrate, PAA-, PMMA- and PDMS-coated SERS sensors, Figure S4: Raman spectra of phenethyl alcohol gas detected by (a) PAA, (b) PMMA and (c) PDMS coated SERS gas sensor, Figure S5: Raman intensity at 1007 cm^−1^ of random 30 points obtained by using (a) PAA, (b) PMMA and (c) PDMS coated sensors for acetophenone gas detection, Figure S6: Raman spectra of acetophenone gas detected by (a) PAA, (b) PMMA and (c) PDMS coated sensors, Figure S7: Raman intensity at 1175 cm^−1^ of random 30 points obtained by using (a) PAA, (b) PMMA and (c) PDMS coated sensors for anethole gas detection, Figure S8: Raman spectra of anethole gas detected by (a) PAA, (b) PMMA and (c) PDMS coated sensors, Figure S9: PCA score plots of phenethyl alcohol, acetophenone and anethole gases detected by bare SERS substrate, Figure S10: PCA score plots of the (a) two one-layer film coated PMMA, PAA, (b) one two-layer film coated PMMA-PAA SERS sensors for phenethyl alcohol, acetophenone and anethol gases, Figure S11: PCA score plots of the (a) two one-layer film coated PDMS, PMMA, (b) one two-layer film coated PDMS-PMMA SERS sensors for phenethyl alcohol, acetophenone and anethol gases.
