# Supplementary material for: SERS Gas Sensors Based on Multiple Polymer Films with High Design Flexibility for Gas Recognition
Source: Sensors (Basel). 2021 Aug 18;21(16):5546. doi: 10.3390/s21165546 (PMC8402296; doi:10.3390/s21165546)

SERS gas sensor based on multiple polymer film with high design flexibility for gas recognition

Lin Chen [1], Hao Guo [1], Fumihiro Sassa [1], Bin Chen [2,*], and Kenshi Hayashi [1,*]

[1] Graduate School and Faculty of Information Science and Electrical Engineering, Kyushu University, Fukuoka, Japan; chen.lin.658@s.kyushu-u.ac.jp (L.C), hayashi@ed.kyushu-u.ac.jp (K.L)

[2] Chongqing Key Laboratory of Non-linear Circuit and Intelligent Information Processing, College of Electronic and Information Engineering, Southwest University, Chongqing, China; chenbin121@swu.edu.cn

* Correspondence: chenbin121@swu.edu.cn (B.C), hayashi@ed.kyushu-u.ac.jp (K.L)

Supplemental Information

Supplemental Figure

Figure S1. (a) SEM images of Ag nanoparticles (b) EDX results of selected area obtained from bare substrate.

Figure S2. Raman spectra of (a) Phenylethyl alcohol, (b) Acetophenone and (c) Anethole solution detected by bare SRES substrate.

Figure S3. The background Raman spectra of bare substrate, PAA-, PMMA- and PDMS-coated SERS sensors.

Figure S4. Raman spectra of phenethyl alcohol gas detected by (a) PAA, (b) PMMA and (c) PDMS coated SERS gas sensor.

Figure S5. Raman intensities at 1007 cm^{-1} of random 30 points obtained by using (a) PAA, (b) PMMA and (c) PDMS coated sensors for acetophenone gas detection.

Figure S6. Raman spectra of acetophenone gas detected by (a) PAA, (b) PMMA and (c) PDMS coated sensors.

Figure S7. Raman intensities at 1175 cm^{-1} of random 30 points obtained by using (a) PAA, (b) PMMA and (c) PDMS coated sensors for anethole gas detection.

Figure S8. Raman spectra of anethole gas detected by (a) PAA, (b) PMMA and (c) PDMS coated sensors.

Figure S9. PCA score plots of phenethyl alcohol, acetophenone and anethole gases detected by bare SERS substrate.

Figure S10. PCA score plots of the (a) two one-layer film coated PMMA, PAA, (b) one two-layer film coated PMMA-PAA SERS sensors for phenethyl alcohol, acetophenone and anethol gases.

Figure S11. PCA score plots of the (a) two one-layer film coated PDMS, PMMA, (b) one two-layer film coated PDMS-PMMA SERS sensors for phenethyl alcohol, acetophenone and anethol gases.

Figure S1.

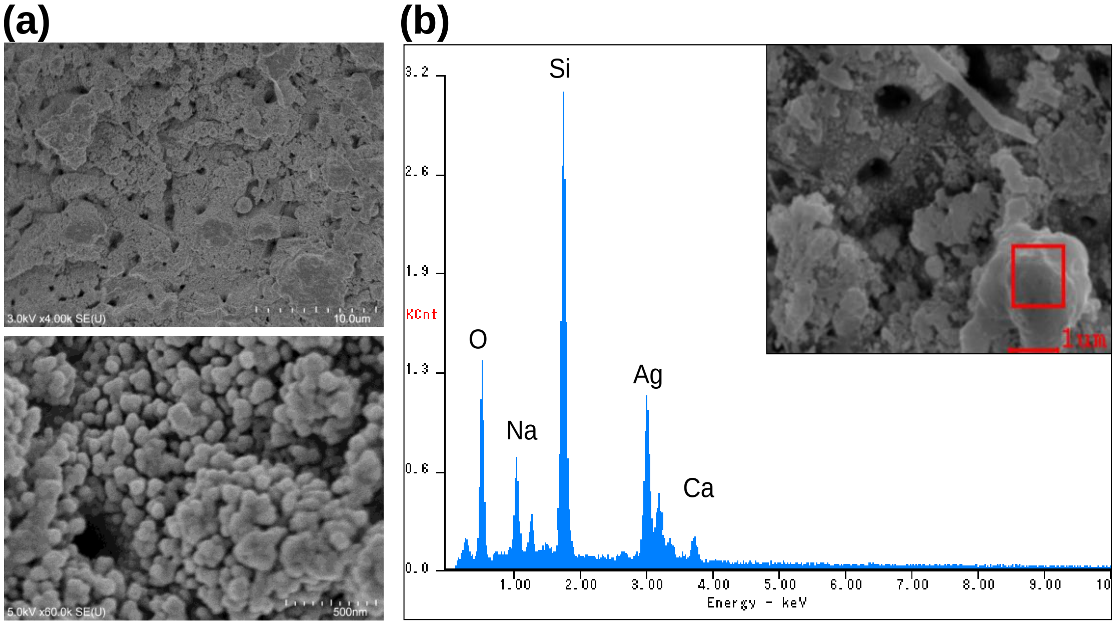

Figure S2.

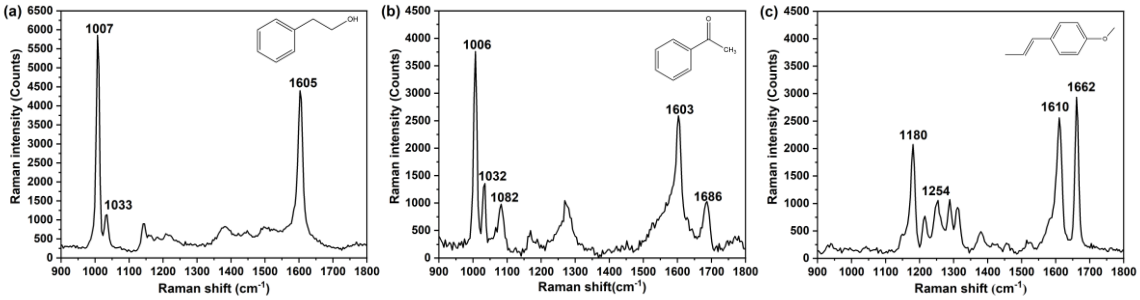

Figure S3.

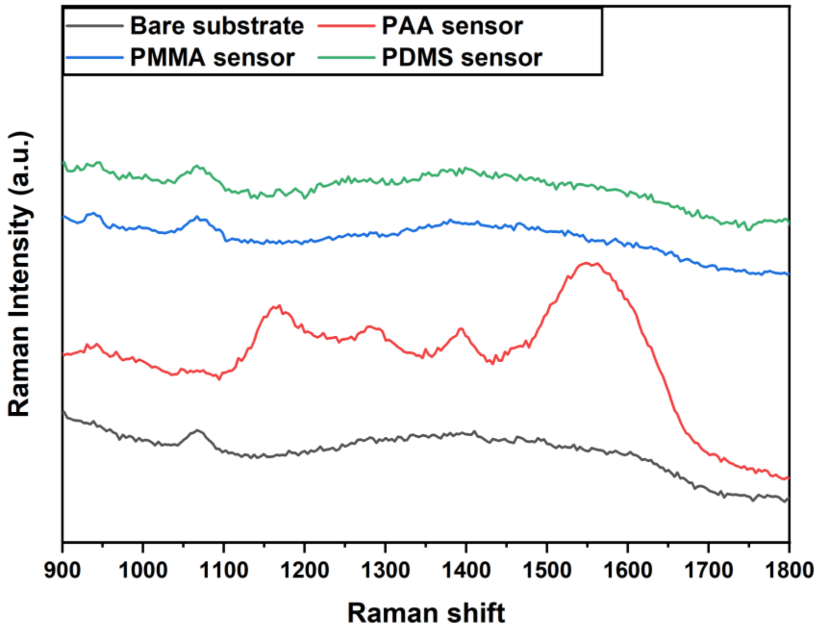

Figure S4.

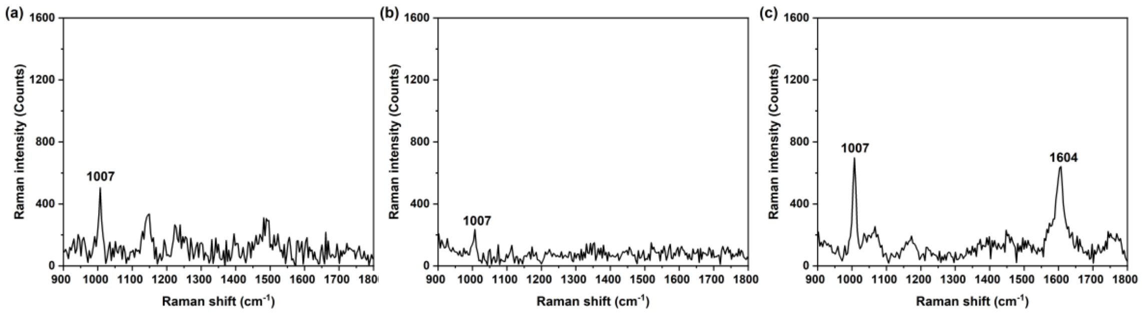

Figure S5.

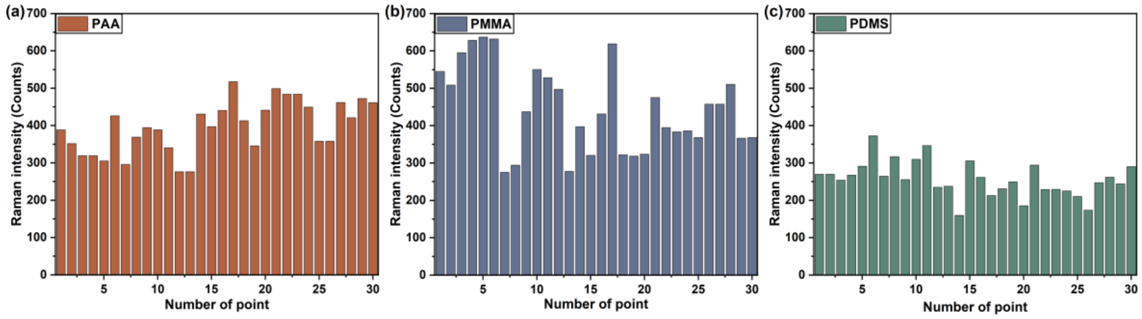

Figure S6.

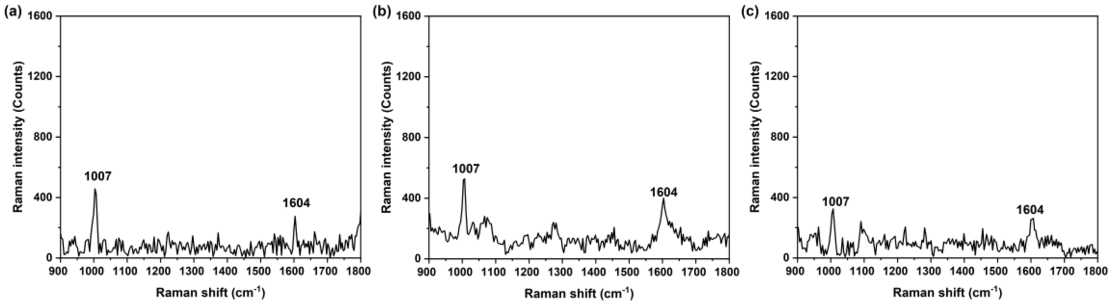

Figure S7.

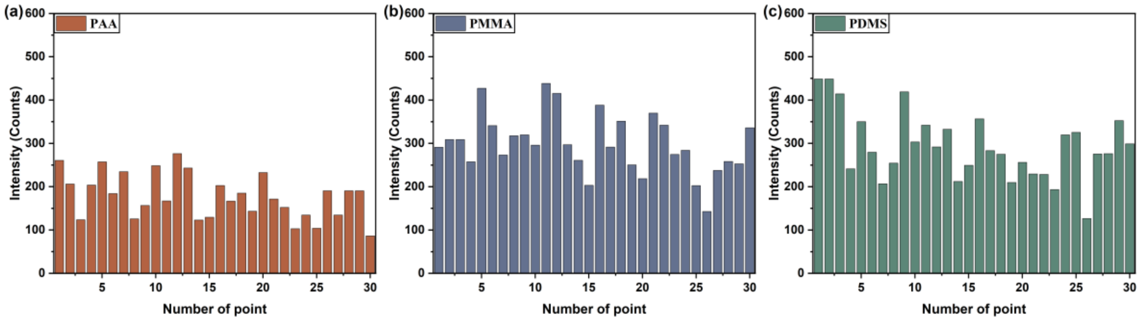

Figure S8.

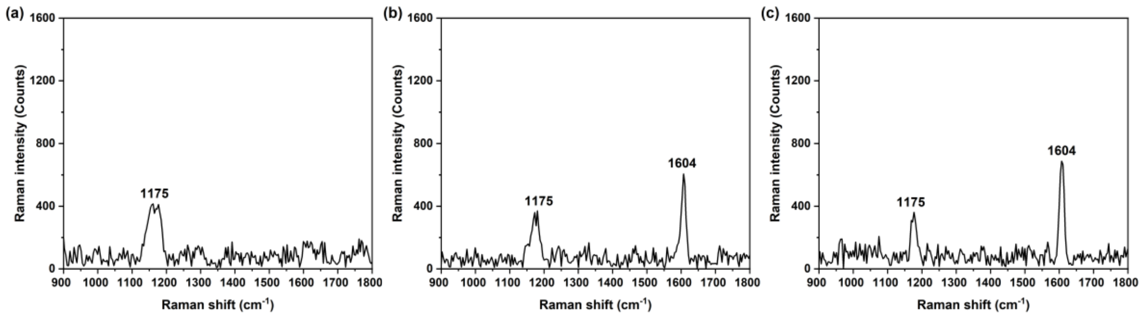

Figure S9.

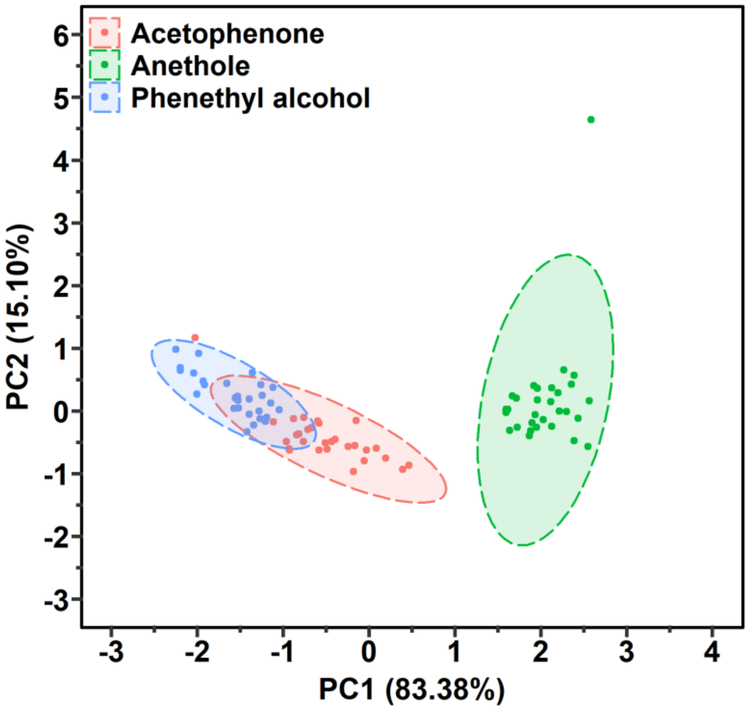

Figure S10.

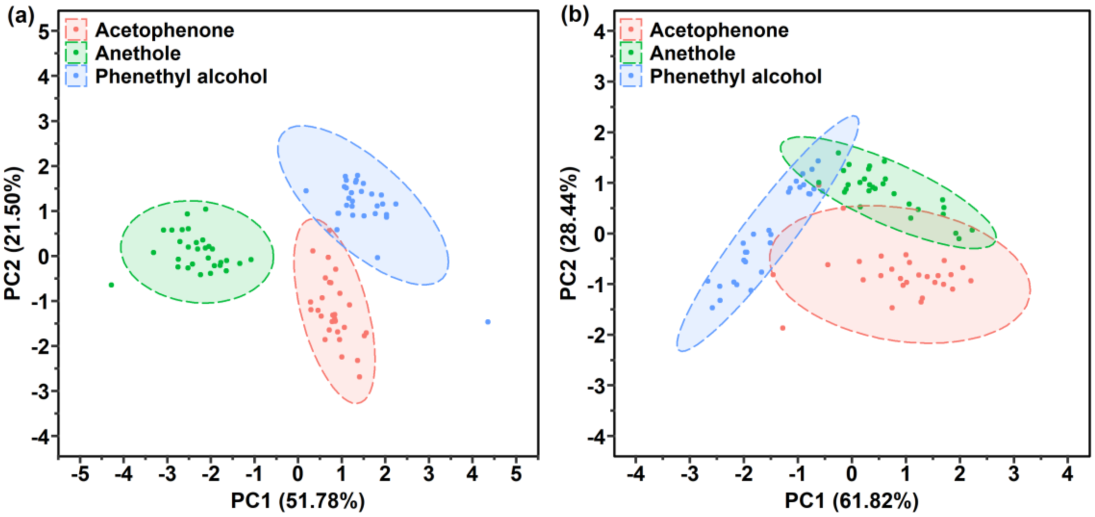

Figure S11.

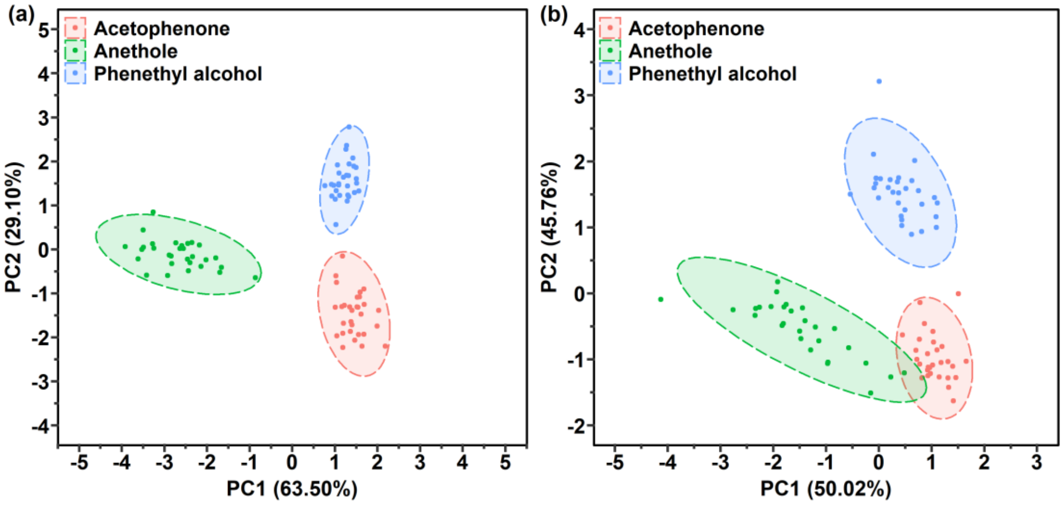

Supplement: Supplementary file 1 [file sensors-21-05546-s001.zip › sensors-1325303-supplementary.pdf]
